# Health Care Professionals’ Experiences of Patient-Professional Communication Over Patient Portals: Systematic Review of Qualitative Studies

**DOI:** 10.2196/21623

**Published:** 2020-12-08

**Authors:** Elina Laukka, Moona Huhtakangas, Tarja Heponiemi, Sari Kujala, Anu-Marja Kaihlanen, Kia Gluschkoff, Outi Kanste

**Affiliations:** 1 Finnish Institute for Health and Welfare Social and Health System Research Unit Helsinki Finland; 2 Research Unit of Nursing Science and Health Management University of Oulu Oulu Finland; 3 Department of Computer Science Aalto University Espoo Finland; 4 Department of Psychology and Logopedics University of Helsinki Helsinki Finland

**Keywords:** telemedicine, communication, patient portals, nurses, physicians, systematic literature review, thematic analysis

## Abstract

**Background:**

The popularity of web-based patient-professional communication over patient portals is constantly increasing. Good patient-professional communication is a prerequisite for high-quality care and patient centeredness. Understanding health care professionals’ experiences of web-based patient-professional communication is important as they play a key role in engaging patients to use portals. More information is needed on how patient-professional communication could be supported by patient portals in health care.

**Objective:**

This systematic review of qualitative studies aims to identify how health care professionals experience web-based patient-professional communication over the patient portals.

**Methods:**

Abstract and full-text reviews were conducted by 2 reviewers independently. A total of 4 databases were used for the study: CINAHL (EBSCO), ProQuest (ABI/INFORM), Scopus, and PubMed. The inclusion criteria for the reviewed studies were as follows: the examination of health care professionals’ experiences, reciprocal communication between patients and health care professionals, peer-reviewed scientific articles, and studies published between 2010 and 2019. The Joanna Briggs Institute’s quality assessment criteria were used in the review process. A total of 13 included studies were analyzed using a thematic synthesis, which was conducted by 3 reviewers.

**Results:**

A total of 6 analytical themes concerning health care professionals’ experiences of web-based patient-professional communication were identified. The themes were related to health care professionals’ work, change in communication over patient portals, patients’ use of patient portals, the suitability of patient portals for communication, the convenience of patient portals for communication, and change in roles.

**Conclusions:**

Health care professionals’ experiences contain both positive and negative insights into web-based patient-professional communication over patient portals. Most commonly, the positive experiences seem to be related to the patients and patient outcomes, such as having better patient engagement. Health care professionals also have negative experiences, for example, web-based patient-professional communication sometimes has deficiencies and has a negative impact on their workload. These negative experiences may be explained by the poor functionality of the patient portals and insufficient training and resources. To reduce health care professionals’ negative experiences of web-based patient-professional communication, their experiences should be taken into account by policy makers, health care organizations, and information technology enterprises when developing patient portals. In addition, more training regarding web-based patient-professional communication and patient portals should be provided to health care professionals.

## Introduction

### Background

Due to the World Health Organization’s aims for health care digitalization and due to the COVID-19 pandemic, the importance of eHealth has increased considerably [1,2]. Internet-based interactive health services such as patient portals represent one form of eHealth [3]. Patient portals are secure websites that offer patients access to a variety of functions, such as viewing laboratory results and secure messaging; these portals are administrated and owned by health care organizations [4,5]. Patient portals provide patients with remote web-based access to their personal health information, services, and clinical care [3], and occasionally patient portals are synchronized with electronic health records (EHRs) [4]; however, they may also be individual web pages with no connection to EHRs. Occasionally, portals enable reciprocal communication between patients and health care professionals [6,7], for example, via secure electronic messaging [8].

Good patient-professional communication is a prerequisite for high-quality care and a key element of patient centeredness [9]. Traditionally, patient-professional communication happened face-to-face during clinical consultations [8]; however, in recent years, communication through patient portals has also become common [10]. Patient portals enable reciprocal communication and interactive guidance and coaching of patients, which may be more effective than just providing patients with clinical information, such as doctors’ notes, without any further advice [4]. Some patients even prefer to use web-based communication, for example, because it can be seen as less intimidating than face-to-face encounters [11,12]. Patients have reported several benefits related to the use of patient portals. For example, patients with diabetes were significantly more likely to believe that reading their doctor’s notes would improve their self-care and adherence to medication [13]. In addition, web-based communication with patients with cancer may have just as big of an impact on their care as face-to-face communication [14]. The portals not only helped patients to better manage their diseases but also conferred psychological benefits, such as increasing trust and collaboration with health care providers [15].

Patient portals provide several benefits for service providers, such as reduced amounts of hospital-based care [16] and cost-effectiveness [17]. However, many physicians have expressed concerns that the use of patient portals could change the patient-professional relationship [18]. In a study by Daniel et al [19], the majority of physicians showed reluctance to use web-based apps and social media to communicate with patients. In addition, some health care professionals have been unwilling to inform patients about patient portals and have expressed concern that patient portals may reduce their professional autonomy [20]. Despite concerns and reluctance of health care professionals, digitalization and web-based communication have already become a part of health care professionals’ expected competency [21].

Traditional face-to-face patient-professional communication has been widely examined [9,22,23], but less attention has been paid to web-based patient-professional communication that occurs over patient portals [24]. Previous studies have shown conflicting results related to web-based patient-professional communication. In a systematic review, Kruse et al [25] found that several positive and negative attributes of web-based patient-professional communication overlapped within the same study. For example, although some patients and professionals perceived an element of a patient portal to be beneficial, other respondents had negative experiences related to the same element in their portal [25]. Another systematic review supports the nature of bifurcation; Ferreira et al [26] found that some studies enhanced patient-professional communication but also showed patients’ concerns about confidentiality and understanding of the content.

Web-based patient-professional communication has been synthesized in a scoping review by Voruganti et al [27], who aimed to map, describe, and understand web-based tools for communication between patients and physicians. They found that web-based tools for patient-professional communication were most prevalent in contexts where the intended use was the patients’ self-management [27]. In this review, the experiences of health care professionals will be scrutinized to gain broader knowledge about web-based patient-professional communication. This review focuses on patient portals, as they seem to be the most commonly used web-based tools for patient-professional communication [27].

Understanding the experiences of health care professionals with patient portals is important because health care professionals play a key role when supporting and engaging patients in their use of these portals [20]. Endorsement of patient portals by health care professionals is one of the most influential factors impacting patients’ use of patient portals as well as their use as tools for collaborative communication [28]. Aligning patient portals with health care professionals’ workflow and care delivery priorities is difficult, and this might impact the professionals’ experiences of the patient-professional communication over patient portals [29]. According to Irizarry et al [29], greater understanding is needed of how patient-professional communication could be supported by patient portals in practical care work.

### Objectives

As previous reviews have found conflicting results [25] and concentrated on web-based tools instead of communication [27], a more detailed understanding is needed on patient-professional communication from a professional perspective. Adding this information may support the use of patient portals in practical care work. In light of these gaps in the research, a systematic literature review was conducted to identify the experiences of health care professionals with web-based patient-professional communication over patient portals. In this study, we define communication as reciprocal web-based communication or interaction between health care professionals and patients. With reciprocal, we mean that patients can answer their health care professional directly using, for example, secure messaging. The following research question was addressed: What kind of experiences do health care professionals have of web-based patient-professional communication over patient portals?

## Methods

### Information Sources and Search Strategy

A systematic review of qualitative studies was conducted following the Joanna Briggs Institute’s (JBI) Reviewer’s Manual [30], including the application of a PRISMA (Preferred Reporting Items for Systematic Reviews and Meta-Analyses) checklist [31]. A systematic review method was chosen for this review because it is applicable in areas where there is only a little preexisting knowledge and where complex issues require more detailed exploration [32]. With the assistance of an experienced information specialist, the searches were conducted in the CINAHL (EBSCO), ProQuest (ABI/INFORM), Scopus, and PubMed databases using search terms related to portals and patient-professional communication (Table 1). A total of 1038 articles were found, which were reduced to 597 after duplicates were removed using the RefWorks Legacy reference management software package.

**Table 1:** Databases, search strategy, and results identifying the studies.

Database	Search strategy	Results, n
Cumulative index to nursing and allied health literature (CINAHL [EBSCO])	ALL^a^(portal OR portals) AND ALL(patient* N5 (professional* OR provider* OR physician* OR doctor* OR nurse*) ) AND ALL(communicat* OR interact*)	183
ProQuest (ABI/INFORM)	ALL(portal OR portals) AND ALL(patient* N/5 (professional* OR provider* OR physician* OR doctor* OR nurse*)) AND ALL(communicat* OR interact*)	12
Scopus	ALL(portal OR portals) AND ALL(patient* W/5 (professional* OR provider* OR physician* OR doctor* OR nurse*) ) AND ALL(communicat* OR interact*)	382
PubMed	TEXT WORD^b^(portal OR portals) AND TEXT WORD(communicat* OR interact*) AND TEXT WORD(patient* AND (professional OR provider OR physician OR doctor* OR nurse*)	461

^a^ALL: Everywhere but the whole text, that is, including the title, abstracts, and keywords.

^b^TEXT WORD: Terms that are qualified with the Text Word field tag will be searched in the following fields: title, abstract, medical subject headings (MeSH) heading and subheadings, other terms field, secondary source identifier, and personal name as subject.

### Study Selection Process

**Figure 1:**
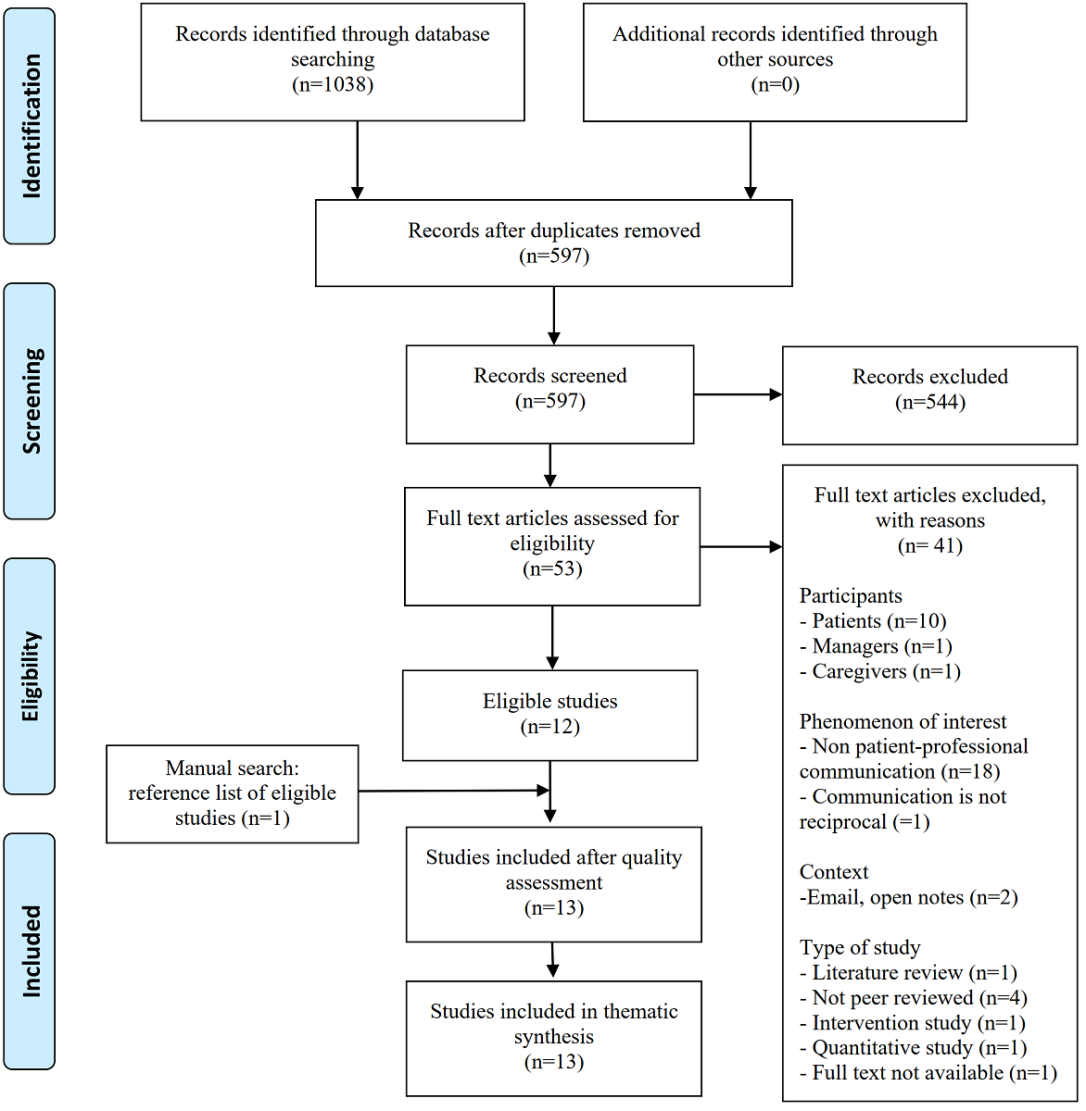
A Preferred Reporting Items for Systematic Reviews and Meta-Analyses flow diagram of the systematic review carried out in this study.

The study selection process is presented in the PRISMA flow diagram shown in Figure 1. The studies were screened by title and abstract (n=597) and the full text (n=53) independently by 2 researchers (EL and MH). The inclusion was based on eligibility criteria that were defined according to the participants, phenomenon of Interest, context, study type method: (1) the *P*articipants were health care professionals; (2) the phenomenon of *I*nterest was communication; (3) the *Co*ntext referred to patient portals; and (4) as a *S*tudy type, only studies including qualitative data were included because they better examined experiences (Table 2). All the studies were published between 2010 and 2019. This time span is relevant for researching patient portals, which are a relatively new phenomenon and are constantly developing. The 2 reviewers discussed and agreed on which studies should be included according to the inclusion criteria. A total of 41 articles were excluded because they did not meet the eligibility criteria. Moreover, 12 of the excluded articles were from the patient, manager, or caregiver perspective. In 19 articles, the phenomenon of interest was not in patient-professional communication, but was focused, for example, on scrutinizing the communication between professionals or the communication was not reciprocal. In addition, 3 articles scrutinized web-based patient-professional communication in emails and open notes instead of patient portals. Finally, 8 studies represented the wrong study type or the full text was not available. The reasons for exclusions are presented in Figure 1. The reference lists of all the included studies (N=13) were manually searched for additional studies (n=1). If any disagreements occurred at any point in the article selection process, they were resolved by consulting the last author of this paper (OK). A kappa value of 0.72 in the title-abstract screening and 0.95 in full-text screening showed a substantial and almost perfect level of agreement [33].

**Table 2:** Eligibility criteria according to the PICoS protocol.

PICoS^a^	Inclusion criteria	Exclusion criteria
Participants	Health care professionals	Other than health care professionals (eg, managers or patients)
Phenomenon of interest	Reciprocal web-based communication or interaction between health care professionals and patients	Communication occurs between health care professionals and communication occurs between health care professional and family member or caregiver
Context	Patient portal	Other than patient portal (eg, email, video consultations, and open notes)
Types and quality of studies	Peer-reviewed scientific studies containing qualitative data, published between 2010 and 2019, full text available	Quantitative studies, gray literature, literature reviews, systematic reviews, study protocols, and intervention studies

^a^PICoS: participants, phenomenon of interest, context, study.

### Quality Assessment

The quality of the studies (N=13) chosen for the review was assessed by 2 independent reviewers (EL and MH) using the JBI checklist for qualitative research [34]. To achieve consensus between the reviewers, the selected studies had to achieve at least a score of 5 out of 10 across the quality criteria [35]. All 13 studies were considered suitable for the review.

### Data Extraction and Synthesis

The data were extracted by the authors, year of publication, country of origin, purpose, participants, methods (data collection and analysis), relevant findings, and quality assessment (Table 3). The thematic synthesis presented by Thomas and Harden [36] was used to synthesize the results. The synthesis included line-by-line coding of the findings, organizing initial codes (n=162) to construct descriptive themes (n=48), the use of subthemes when necessary to clarify the reporting of the results (n=11), and finally the development of analytical themes (n=6) [36,37]. An example of coding and theme building is presented in Table 4.

**Table 3:** Data extraction of the included studies.

Reference, country of origin	Purpose	Participants (relevant participants for the review)	Methods (data collection and analysis)	Relevant findings	Quality assessment points
Alpert et al [38], United States	To evaluate how well portals convey information to patients. To demonstrate how methodologies could be used to evaluate and improve the design of portals.	Clinicians (n=13), patients (n=31).	Interviews of patients, focus groups for clinicians (n=2). Thematic analysis.	Clinicians believed that the portal was not well equipped to handle complex communication. They found it complicated that there was no confirmation that the patients viewed the messages. Asynchronous communication patterns disrupted care.	7/10
Alpert et al [39], United States	To describe the types of messages initiated by patients communicating via patient portals and to assess whether providers employed patient-centered strategies in their responses.	N/A^a^	A total of 193 messages from 58 message threads. Content analysis.	Providers limited their responses strictly to the requested information, and the majority of their responses lacked empathy. Occasionally, providers adopted a “customer service-oriented” approach. Some of the providers’ messages reinforced positive patient behavior.	6/10
Alpert et al [40], United States	To understand attitudes of the portal’s adoption for oncology and to identify the advantages and disadvantages of using the portal to communicate and view medical information.	Oncologists (n=13), medical informaticists (n=12), and patients (n=35).	In-depth semistructured interviews. Thematic text analysis.	Oncologists believed that advanced access may improve engagement during consultations and also assist patients after appointments.	7/10
Alpert et al [41], United States	To understand the perceptions of oncologists and cancer patients about the potential impact of portals on such communication.	13 oncologists and 35 patients.	In-depth, semistructured interviews. Thematic analysis.	Oncologists agreed that patient access to their records was beneficial, but they were also concerned about the workload and portal not being suitable for complex information. Oncologists were also concerned that patients would anticipate rapid communication and that they would learn about a new diagnosis before meeting the oncologist.	8/10
Bishop et al [42], United States	To answer the following research questions: (1) how can primary care practices use electronic communication to manage clinical issues; (2) what are the perceived advantages and disadvantages of these programs for patients, physicians, and practices; and (3) what are the barriers to and facilitators of implementation of electronic communication programs?	Leaders of 21 medical groups and also staff in 6 of these groups.	Interviews analyzed using a constant comparative method.	Frontline physicians agreed that electronic communication improved access to care for patients, saved patients’ time, and improved patient satisfaction. They also cited efficiency as an advantage of patient portals. Physicians were able to contact patients before the consultations, which improved the efficiency of office visits. Increased workload and patient and physician resistance were identified as disadvantages.	6/10
Das et al [43], Norway	To characterize and assess the impact of an eHealth portal on health care professionals’ interaction with patients in bariatric surgery.	Health care professionals (n=3).	Semistructured in-depth interviews that were thematically analyzed.	By following the patients’ writing, the professionals learned more about their patients than during time-limited face-to-face consultations. The portal became an important way to reach out for the patients. Yet, professionals reported uncertainty about how to deal with new kinds of interaction, and they were not able to ensure that patients understood the given information. The portals also increased the workload, interfered with the workflow, and were not suitable for complex cases.	7/10
Elers and Nelson [44], New Zealand	To identify how general practitioners perceive patient portals to influence the delivery of primary health care.	General practitioners (n=9).	Semistructured interviews that were thematically analyzed.	General practitioners expressed that the internet was just another way to interact with the patients.	7/10
Gerber et al [45], United States	To identify nursing staff reactions to and perceptions of electronic portal use in a cancer setting.	Outpatient nurses (n=13).	Focus groups (n=2). Theoretical thematic content analysis.	Nurses were concerned about the increase in the volume of electronic communications and that patients expected immediate responses.	8/10
Kopanitsa [46], Russia	To analyze the attitudes of patients with tuberculosis and doctors and identify perceived opportunities and barriers to operate a web portal.	General practitioners (n=10), phthisiatricians (n=8), and patients (n=30).	Semistructured interviews. Grounded theory and thematic analysis.	Doctors reported that the web portal would improve their communication with patients, but should not create any additional work.	8/10
Miller et al [47], United States	To determine how administrators, clinic staff, and health care providers at a practice serving a lower-income adult population viewed patient portals in terms of their potential benefit, areas of concerns, and hopes for the future.	Clinical personnel (n=20).	In-depth interviews. Data analysis was based on a systematic, computer-assisted approach.	Nurses, physicians, and clinic personnel agreed that electronic messaging was quicker and more efficient. Several nurses and physicians were worried that some patients would inappropriately send repeated messages and may expect immediate responses to their electronic requests. Health care professionals were aware that patients saw all the posts.	7/10
Nazi [48], United States	To examine the experiences of physicians, nurses, and pharmacists at the Department of Veterans Affairs using an organizationally sponsored personal health records to develop insights into the interaction of technology and processes of health care delivery.	Health care professionals (n=30).	In-depth interviews. Utilized modern techniques of qualitative analysis.	Secure messaging enabled better connectivity between patients and their health care teams. Asynchrony was seen as a benefit that allowed health care professionals to send and respond to messages when it was convenient for them. Secure messaging enabled health care professionals to better know their patients and enhanced the quality of visits.	7/10
Sieck et al [49], United States	Within primary care offices with high rates of patient portal use, the study examined how experienced physicians and patient users of the ambulatory portal perceived the benefits and challenges of portal use in general and secure messaging in particular.	Primary care physicians (n=13) and patients (n=29).	Semistructured interviews. Both inductive and deductive methods, using a constant comparative analytic approach.	Providers noted increased efficiency in communications, but some of the problems were too complex to handle via secure messaging. Sometimes the providers noted that patient messages did not contain enough information, and hence, they were worried about the quality of the provided information.	8/10
Vreugdenhil et al [50], Netherlands	To explore the adoption, use, usability, and usefulness of a recently introduced patient portal in an academic hospital to learn lessons for the implementation of patient portals in fragmented health care systems.	Medical specialists (n=3), medical specialists in training (n=4), nurses (n=4), administrative assistants (n=3), doctor’s assistant (n=1), managers (n=2), and patients (focus group n=7, think-aloud observation n=8).	Focus groups (n = 4) and think-aloud observations for patients. Thematic content analysis.	Not all the health care professionals agreed on how the messaging functionality should be used. Some doctors preferred not to use messaging functionality to answer questions, especially when it concerned complex problems. Health care professionals felt they lost some control because of the portal.	7/10

^a^N/A: not applicable.

**Table 4:** An example of coding in thematic synthesis.

Line-by-line coding^a^	Initial code^b^	Descriptive theme^c^	Subtheme^d^	Analytical theme^e^
“It is just that the days are filled with patient lists, and suddenly it is 4 o’clock, and then you are off to home. We haven’t organized the time for it...” [43]	No time organized for portal communication	Not enough time resources	Lack of time and expertise for portal communication	Health care professionals’ work
“The way my day is set up right now, I am scheduled to see patients; I really have no time to respond...” [42]	No time to respond	—^f^	—	—
“You need to plan extra time to process these messages.” [50]	Need for extra time	—	—	—
“’Linda’ explained that the activities triggered by this one question required considerable effort: the process required resources in regards to expertise in knowing the right addressee, time and effort to contact them...” [43]	Time effort	—	—	—
“...time constraints, and prioritizations became evident in the daily clinical practice...” [43]	Time constraints	—	—	—
“This represented a challenge for the level of expertise required...” [43]	Challenges due to the level of expertise required	Lack of expertise	—	—
“In cases when the personnel with portal access could not respond themselves, they made contact with other professionals at the clinic...” [43]	Professionals could not answer themselves	—	—	—

^a^Free line-by-line coding of the findings of primary studies.

^b^Initial codes based on line-by-line coding and formed into a *bank* of codes.

^c^Initial codes categorized as descriptive themes.

^d^Descriptive themes were grouped under a subtheme.

^e^Sufficiently abstract analytical themes were created to describe all the descriptive themes. This table does not contain all the descriptive themes that were categorized under Health care professionals’ work.

^f^The empty cells are not supposed to contain text.

First, free line-by-line coding was performed for the findings of the primary studies. Second, initial codes were created using line-by-line coding. Every sentence had at least one initial code, but using several codes for a sentence was also possible, albeit rare, in this review. The use of line-by-line coding enabled the translation of concepts from one study to another. Initial codes formed a *bank* of codes, and new codes were developed when necessary, and the synthesis began at this point. Third, codes were grouped into descriptive themes based on their similarity. Heretofore, the synthesis was kept very close to the original findings of the included studies. Fourth, some of the descriptive themes were grouped under subthemes. Finally, analytical themes were formed. After this phase, the analytical themes were sufficiently abstract to describe all the initial descriptive themes (Table 5) [36].

**Table 5:** An overview of analytical themes, subthemes, and descriptive themes (number of studies out of the included 13).

Analytical themes (n=6)	Subthemes (11)	Descriptive themes (n=48)
Health care professionals’ work (13)	Increased workload	Increases workload (8) Causes more steps in the care process (6) Causes additional tasks (1)
	More efficient work	Increases efficiency of work (6) Saves time (2) Reduces telephone communication (2)
	Experiences of fear and discomfort	Causes fear (3) Confusion in professional activities (2) Uncertainty about portal communication (1) Increases pressure to contact patients faster who actively use the portal (1)
	Increased awareness of the patients’ situations	Provides more information about the patients (3) Enables better overall impression of the patients’ situations (2)
	Lack of time and expertise for engaging in portal communication	Not enough time or resources (3)
Change in communication over the patient portals (12)	Enhanced communication	Enables more direct communication (6) Enhances connectivity (2) Promotes more frequent communication (2) More focused attention (2) Easy way to communicate (2) Safe means of communication (2) Improves communication and relationship (1)
	Change in the means of communication	Transforms the means of communication (5) Variable nature of communication (1)
	Deficiencies in communication	Inability to communicate appropriately (4) Insensitive communication (2) General nature of information, not detailed (2)
Patients’ use of patient portals (12)	Interpretation and communication	Uncertainty about whether patients understand the information (8) Patients communicating inappropriately (3) Patients providing insufficient information (1)
	Positive consequences for patients	Enables patient outcomes and experiences (6) Promotes patients’ involvement (4) Lowers threshold of communication (2) Nonstigmatic way to communicate (1) Patients ask more specific questions (1)
	Patients’ high expectations of professionals	Patients’ expectations for rapid communication (3) Patients’ expectations for use of data (1)
Suitability of the patient portals for communication (7)	N/A^a^	Not suitable for complex communication (3) Not suitable for complex cases (3) Useful in remotely managing patients’ conditions (2) Not suitable for complex communication (1) Uniqueness of patient cases (1)
Convenience of the patient portals for communication (7)	N/A	Flexibility to answer when convenient (4) Possibility to asynchronous communication (3) Portal seen as a nuisance (3) Uncertainty if patients receive the information (1)
Change in roles (4)	N/A	Changes patients’ roles (2) Maintains health care professionals’ responsibility for the patients (2) Transforms health care professionals’ roles (1)

^a^N/A: not applicable.

The synthesis was conducted independently by 3 reviewers (EL, MH, and OK) at all stages. After each stage, the reviewers looked for similarities and differences in their codes and themes and agreed on the final versions.

## Results

### Study Characteristics

The included studies (N=13) originated from the United States (n=9, 69%), Norway (n=1, 8%), the Netherlands (n=1, 8%), New Zealand (n=1, 8%), and Russia (n=1, 8%). The informants in the included studies were reported to be physicians (n=82), health care professionals or clinical personnel (n=63), nurses (n=17), and medical specialists in training (n=4). The number of informants was not reported in 2 studies, but it was reported that they were health care professionals. The data were most commonly collected using individual interviews (n=10), but focus groups (n=2), message threads (n=1), and observation (n=1) were also used for collecting data.

### Health Care Professionals’ Experiences of Web-Based Patient-Professional Communication Over Patient Portals

When analyzing the included studies (N=13), 6 analytical themes were identified that were experiences related to (1) the health care professionals’ work (13/13, 100%), (2) changes in communication over the patient portals (12/13, 92%), (3) the patients’ use of patient portals (12/13, 92%), (4) the suitability of patient portals for communication (7/13, 54%), (5) the convenience of the patient portals for communication (7/13, 54%), and (6) the change in roles (4/13, 8%; Table 5).

#### Experiences Related to Health Care Professionals’ Work

Experiences related to health care professionals’ work were divided into 5 subthemes, which related to the increased workload [39,41,42,44-48,50], more efficient work [42,44,47,48,50,51], experiences of fear and discomfort [41,43,46,49,50], increased awareness of patients’ situations [41,43,48], and lack of time and expertise required for portal communication [42,43,50].

##### Increased Workload

The health care professionals in the studies were concerned about the patient-professional communication increasing their workload [41,42,44,46,48,50]. Although they saw patient participation as a positive outcome of portal communication, having greater patient participation increased the workload [41]. Sometimes health care professionals received messages from patients who did not actually need their help [46], and some of the patients overwhelmed the health care professionals by inappropriately sending them repeated messages [47]. There were organizations where answering the portal messages was not formally incorporated into the daily work process of the professionals, and thus, some of the health care professionals had multiple portal messages waiting for responses, causing them an additional workload [43,50].

Web-based patient-professional communication also increased the steps in the care process [38-40,43,45,47]. For example, web-based communication sometimes triggered more phone calls and several follow-up questions [38,47]. Thus, health care professionals occasionally found it easier to just recommend that the patients scheduled an appointment instead of communicating on the internet [39]. Health care professionals could not always trust that the patient had truly received the information over the patient portal [38]. Thus, the professionals had to ensure by phone that patients really had received the information [38]. When answering the patients, health care professionals also felt a need to ensure that the content of their message was correct and that the patient would not misunderstand the core message [43]. This led to another extra step in the process.

##### More Efficient Work

Some health care professionals reported that web-based patient-professional communication increased the efficiency of their work [42,44,47,48,50,51]. Not only was the web-based communication itself more effective but it also improved the efficiency and quality of face-to-face office visits because patients were able to communicate with professionals before the visits [42,47,48,50]. Face-to-face visits were improved because communicating with the patients on the internet before the visits helped the professionals to be more prepared for the consultation [42]. According to some health care professionals, communicating over the portal also saves time because sending electronic messages is faster than making phone calls [47]. Due to the web-based patient-professional communication, health care professionals were able to reduce the volume of incoming phone calls [47], thus avoiding some challenges encountered with them [48].

##### Experiences of Fear and Discomfort

In some cases, communicating over the patient portals caused a degree of fear and concern among health care professionals. Health care professionals feared that patients may discover a significant change in their well-being, diagnosis, or prognosis in the doctors’ notes without first communicating with health care professionals [41,46]. Due to this fear, the professionals felt pressure to contact patients who actively used portals faster to prevent them from discovering any changes by themselves [41].

In some cases, health care professionals were afraid of communicating on the internet [43]. Patient portals caused some confusion in the professionals’ activities [49,50] by making health care professionals feel slightly uncomfortable [50] or *getting lost* in their professional activities [49]. In addition, health care professionals felt uncertain about how to deal with portal communication, and they felt that they had to focus on the articulation and content of their messaging, which again required considerable effort [43].

##### Increased Awareness of Patients’ Situations

Web-based patient-professional communication increased the health care professionals’ knowledge of their patients [41] and resulted in receiving more detailed patient information [43]. On patient portals, health care professionals could better capture things that did not come up during face-to-face consultations [43], and patients were more inclined to share sensitive information [48]. Due to web-based patient-professional communication, health care professionals gained a better overall impression of their patients’ situations [43,48]. Due to the more frequent communication, health care professionals knew their patients better [48] and learned more about their patients’ needs [43].

##### Lack of Time and Expertise for Engaging in Portal Communication

Sometimes, health care professionals reported that they did not have enough time for portal communication [42,43,50], and they needed extra time to process the portal messages [50]. For example, some health care professionals answered messages in their spare time [50]. The professionals were also challenged by the level of expertise required for portal communication, as they were not always able to answer portal messages by themselves and needed support from other professionals [43].

#### Experiences Related to Changes in Communication Over Patient Portals

The health care professionals’ experiences were also related to changes in communication over the patient portals. According to professionals, web-based patient-professional communication enhanced communication [39,42,43,46,48,49], but also changed the way of communicating [38,43,44,50], and sometimes led to deficiencies in communication [38,39,45,49].

##### Enhanced Communication

Several descriptive themes showed that web-based patient portals enhanced communication. When communicating on the internet, health care professionals provided patients with more direct answers [39,42,46,48,49] by keeping their writing short and concise [43,48]. Communicating on the internet also increased connectivity [48] and gave health care professionals an additional way to reach out to the patients [43]. Communication over the portal was more frequent, and occasionally [48], the patients’ postings acted as triggers for further communication [43]. Sometimes, due to web-based communication, health care professionals were able to pay more focused attention to their patients’ needs [48]. Communicating on the internet was perceived as easy because it enabled the professions to give direct answers to their patients without using intermediates, while also improving some patients’ access to health services [43,48]. In addition, web-based communication was perceived to be safer than traditional face-to-face communication because portal communication allowed the patients to consider issues that had been discussed earlier [40,42] and it left *a trail* concerning the issues that had been discussed [42].

##### Change in the Means of Communication

Web-based patient-professional communication meant that health care professionals learned to communicate with patients in a new way [43,44]. When communicating on the internet, the dynamics of communication changed [50]. For example, there were no nonverbal clues [43]. Some health care professionals learned to communicate over the portal in a casual way, as they did on the internet in their personal lives [43], and they learned when to use web-based communication instead of other means of communication [38].

##### Deficiencies in Communication

Sometimes health care professionals were unable to communicate appropriately over the portal [38,39,45,49], and in the worst cases, the communication was insensitive [38], lacking in empathy, and overlooked the patients’ cues of distress [39,40]. Insensitive communication did not encourage patients to attempt further communication [39], and in some cases, health care professionals mentioned that they selected a portal function to prevent patients from replying to message threads over the patient portal [45].

Sometimes communication was poor because health care professionals only had limited information available about the patients. For this reason, health care professionals were not always able to respond in a detailed manner, providing less specific advice to the patient [43]. In addition, health care professionals had variations in their expectations and attitudes toward portal communication. For example, although some professionals offered clear guidance over the portals, others were more equivocal [49].

#### Experiences Related to the Patients’ Use of Patient Portals

Health care professionals also had experiences related to the patients’ use of patient portals. Health care professionals were worried about the patients’ interpretations and communication over the portal [39-41,43,44,46,47,49,50], but they also saw that web-based patient-professional communication had positive consequences for patients [39-43,46,48,50]. Furthermore, the possibility of communicating on the internet made health care professionals feel that the patients had high expectations from them [41,45,47,48].

##### Interpretation and Communication

Health care professionals were uncertain whether the patients understood the information that was communicated over the patient portals [43,46,50]. Some health care professionals reported that some of the patients were not able to understand the given information [41,44,47,50] and that the patients made their own interpretations [40]. Not understanding the given information left patients unsure about what to do next [39].

In some cases, patients used the portal inappropriately [49] and sent unsuitable messages to health care professionals [43,47,49]. For example, one patient sent a health care professional a picture from his vacation, which had nothing to do with the patient’s health status [49]. In addition, some patients provided insufficient [49] and poor-quality information [49,50]. For example, patients occasionally write lengthy descriptions devoid of clarity and clear questions [49].

##### Positive Consequences for Patients

From the viewpoint of health care professionals, web-based patient-professional communication had positive consequences for patients, such as better patient engagement [40,41,48], positive patient behavior [39], increased health status [46], and increased trust and satisfaction [48]. In addition, patients were better informed about their diseases and treatments [50] and started to follow the health care professionals’ recommendations more carefully [46]. As the patients had better knowledge, they were able to present more specific questions to health care professionals [43]. Occasionally, the level of their questions was so advanced that the health care professionals felt unprepared for them [43]. Portal communication also promoted patients’ involvement and participation, and the health care professionals asked for their patients’ opinions and concerns over the portal [39,42]. The threshold to discussing fearful or shameful topics was lowered as the negative feelings associated with these topics did not influence communication as much as they would in face-to-face settings [43,48].

##### Patients’ High Expectations of Professionals

When communicating over the portal, health care professionals reported that their patients expected rapid communication [41] and immediate responses to their electronic requests or status updates [45,47]. In addition, the professionals expressed concerns that patients often expected that professionals could view data, for example, on blood glucose levels, they entered, further influencing health care professionals’ endorsement of use [48].

#### Experiences Related to the Suitability of Patient Portals for Communication

The suitability of web-based patient-professional communication varied between patient cases. Patient portals were perceived as useful in some patients’ cases, such as for managing chronic conditions remotely [49] and learning about acute changes in their conditions [46]. However, as each patient’s case was unique [40], communicating over the portal was not the right choice for all cases. Health care professionals experienced difficulties with complex issues [38,50], complex patient cases [49,50], and complex information [41]. According to health care professionals, patient portals were not suitable for communication of complex issues [38,50], and occasionally, they felt that a richer communication platform was needed for more meaningful conversations [38], such as treatment and prognosis discussions [41]. Health care professionals prefer not to use patient portals for complex patient cases [49,50] due to limitations of the portals to convey deeper knowledge [43]. The professionals were also concerned about sharing complex information over the portal and agreed that sharing certain information, such as treatment discussions, was acceptable over the portal, whereas other types of information, such as prognoses, were not suitable for portal communication [41].

#### Experiences Related to the Convenience of Patient Portals for Communication

Some health care professionals appreciated the flexibility to answer portal messages when convenient [43,48,49] and saw asynchronous communication to be beneficial [43,46,48]. Sometimes, the professionals preferred the slower time line of web-based communication, as it allowed them to discuss and research their responses before sending an answer to the patients’ questions [45].

Not all health care professionals preferred web-based patient-professional communication. Some of the health care professionals saw portal communication as a nuisance [42] and felt that asynchrony disrupted care [38]. Poor convenience was also related to communication in those cases when health care professionals did not receive a confirmation that the patient had viewed a message [38]. This left the health care professionals unsure about whether the patients had read their instructions. Health care professionals also had differing views on the messaging functionality of patient portals [50]. For instance, nurses reported that they advised patients to ask questions over the portal, whereas some doctors preferred not to use the portal’s messaging function to answer questions [50].

#### Experiences Related to Changes in Roles

Web-based patient-professional communication has changed the roles of patients and health care professionals [50]. Some professionals reported that communicating with patients on the internet gave the patients more ownership and made them partners of the health care professionals [41,50], which again transformed the professionals’ roles [50]. However, despite the change in their role, the core element of the patient-professional relationship remained the same because after all, the health care professionals were still responsible for their patients [40,42].

## Discussion

### Principal Findings

This systematic review identified how health care professionals experienced web-based patient-professional communication via patient portals. A thematic synthesis produced 6 analytical themes that described health care professionals’ experiences concerning (1) health care professionals’ work, (2) changes in communication over the patient portal, (3) patients’ use of the patient portals, (4) the suitability of the patient portals, (5) the convenience of patient portals, and (6) changes in roles. The descriptive themes described in the analytical themes can be divided into positive and negative experiences, and some of the experiences seem to overlap so that even within the same study, some health care professionals seemed to experience the same feature positively, whereas others experienced it negatively.

Overall, health care professionals experienced web-based patient-professional communication positively, as it made their work more efficient, increased their knowledge about their patients’ situations, enhanced communication, had positive consequences for patients, and changed health care professionals’ and patients’ roles in a positive way. In addition, the findings of prior studies support some of the positive experiences found in this review [6,14,48]. In the review by Otte-Trojel et al [8], several studies mentioned how patient portals enabled building an ongoing, personal relationship that included mutual trust and responsibility. In this review, it seemed that web-based patient-professional communication is especially beneficial in that it produces positive consequences for patients, such as better patient engagement [35-39,42,44,46]. The findings of this review are in agreement with those of the earlier studies, which indicated that web-based communication may drive patient engagement to a new improved level [14,48,49].

In this review, health care professionals also reported that web-based patient-professional communication had a positive impact on health care professionals’ and patients’ roles [37,46] and that the patient-professional relationship remained the same as when having face-to-face contacts [40,42]. Prior studies have shown that health care professionals were worried that web-based communication may change patient-professional relationships [14,50]. In the study by Geerts et al [18], health care professionals expressed concerns that if they did not respond to a digital conversation quickly enough, the patient-professional relationship may change.

In this review, health care professionals’ negative experiences were related to increased workload, time pressure, lack of expertise, communication problems, and the patients’ interpretations and high expectations. In prior studies, the daily workload of health care professionals has also been reported to have a negative impact on web-based patient-professional communication [5,14,51]. In this review, health care professionals were worried about their patients’ ability to understand information given over the patient portals. In the study by Baudendistel et al [52], health care professionals were likewise concerned about patients autonomously handling the information.

The analytical themes of (1) experiences of convenience and (2) changes in the means of communication included both positive and negative aspects. For example, some health care professionals appreciated the flexibility to communicate over the patient portal asynchronously [39,44,45], whereas some saw asynchronous web-based patient-professional communication as a nuisance [42]. In addition, some health care professionals felt that it was because of the portals that they had learned to communicate in a modern way, and they saw this as a positive thing [39,40]. However, some health care professionals also reported that the lack of nonverbal cues, such as body language, tone of voice, and gaze, made it difficult for them to assess whether the patient actually understood the information that they were providing [43].

Some of the included studies seemed to suggest an overall more positive insight into web-based patient-professional communication than other studies. Moreover, in some studies, health care professionals had overlapping positive and negative experiences about patient-professional communication. These kinds of overlapping experiences have also been detected in earlier studies [9,21]. There are a number of possible explanations as to why health care professionals in some studies have more positive experiences with web-based patient-professional communication than the others. First, within the same study, the differences might be explained by variations in the level of digital competence between health care professionals [53]. Those professionals who are more experienced in information technology usage might experience web-based patient-professional communication more positively because they know how to use patient portals and understand when to use them instead of other means of communication [38]. Then, professionals who are not very experienced users of patient portals might struggle with web-based communication and communicate in an inappropriate manner [34,35,41,45].

Second, differences in the experiences of health care professionals in different studies might be attributed to the differences in patient portals. Some of the portals might be less user-friendly than others. According to Kruse et al [25], patient portals obtain a higher level of acceptance if they are user-friendly. Alpert et al [38] noted that health care professionals were concerned about not receiving confirmations or notifications of whether their patients had checked their secure messages; thus, they had to contact patients by phone to ensure that the patients had received their messages. This indicates that the patient portal used in the study was not very user-friendly, as it eventually increased the health care professionals’ workload and required an extra step in the care process [38].

Third, different health care organizations seem to have different practices in providing time and other resources for portal communication. In the study by Das et al [43], there was no time scheduled for web-based patient-professional communication; however, Nazi et al [48] noted that although health care professionals were worried about an increase in their workloads, it had been manageable because the use of the patient portal had grown in an organized way.

This systematic review showed that some health care professionals had learned how to deal with portal communications and identify when web-based patient-professional communication was appropriate [38]. However, some health care professionals seemed to struggle with web-based patient-professional communication, and this may even lead to feelings of fear and discomfort. In addition, a prior study reported that health care professionals found it difficult to learn new ways to communicate with patients over the patient portals [54], and it has also been reported that new skills are required to meet the new demands in the era of eHealth [55].

Health care professionals’ web-based communication skills should be enforced by training because it has been proven to be effective in changing their behavior [14], such as improving the use of core communication skills [7,52] and enhancing empathic expression [56]. In addition, providing health care professionals with more training and technical support on patient portals might be beneficial to ensure positive staff attitudes [57]. More training for health care professionals on this topic might reduce their fear and discomfort concerning communicating over patient portals and assist them in communicating in an appropriate manner. In addition, training could possibly help health care professionals learn about new ways to counsel patients. In the study by Björk et al [58], physicians became more aware of how to communicate with their patients on the internet, how to simplify medical terms, and how to provide extensive medical information after using an Ask the Doctor service.

It appears that web-based patient-professional communication is not suitable for all kinds of communication, such as communicating about complex issues [34,46], complex patient cases [45,46], and complex information [41]. This kind of communication still requires face-to-face consultation or at least contact over the phone. However, in some cases, portal communication may be useful, and sometimes patients are even willing to pay for the possibility of contacting their health care professionals on the internet [59]. For example, web-based patient-professional communication has been seen to be useful in acutely changing conditions [46] and in cases where it is less fearful and shameful to discuss matters on the internet [39,44]. Currently, in the era of the COVID-19 pandemic, some patients display valiant acts of benevolence by preferring remote communication over face-to-face consultations to protect health care professionals from the virus [60]. Using telemedicine, such as patient portals, enables health care organizations to provide care and support for those who require it by minimizing the risk of exposure to patients and health care workers [60]. Due to the COVID-19 pandemic, the use of web-based patient-professional communication may increase considerably and quite rapidly.

### Practical Implications and Further Research

As web-based patient-professional communication is becoming more common [14], more attention should be paid to it by policy makers, health care organizations, and educational institutions. Policy makers aiming for more patient-centered care should understand that developing health care professionals’ web-based communication skills is essential for developing patient-centered health services, and attention should be paid to this issue in health programs and eHealth strategies. Health care organizations should invest in patient portals that are easy to use and functional. For example, they should provide notifications when patients read a message and ensure successful teamwork between professionals. In addition, having multimodal training materials available at sign-up and first portal log-in might be beneficial [61]. Moreover, organizations should schedule enough time for portal communication and arrange training on their use for health care professionals. According to a recent study by Hefner et al [61], training professionals could help them to communicate more efficiently on patient portals, for example, using secure messaging. Health care organizations and enterprises responsible for developing patient portals should also take into consideration the observations of health care professionals about the problems and benefits of patient portals. Health care professionals should also be included in the development of patient portals. Finally, educational institutes should take into account the increase in web-based patient-professional communication in their curricula.

As this review has shown, health care professionals have concerns over their patients’ ability to understand the information provided over patient portals. In future research, it would also be interesting to examine whether patients agree with this concern. Due to the COVID-19 pandemic, the use of patient portals might increase, further transforming the nature of communication and making it an even more essential part of health care. Examining whether a change occurs in attitudes and experiences after the pandemic would also be interesting to provide some very current and up-to-date information on web-based patient-professional communication.

### Limitations

The limitations of this review concern the search strategy, eligibility criteria, and heterogeneity of the selected studies. An electronic search of databases is effective, but it may not identify all eligible studies [62]. For example, only peer-reviewed studies were included in this review, and thus, relevant studies classified as gray literature might have been excluded [30]. This review only included studies that solely considered patient portals. Health care professionals also use other communication forms, such as video consultations [63], which were not considered in this review. Including studies examining video consultations might have provided more detailed information about remote communication; however, communication via video or text might also differ.

The studies included in this review originated from several different countries in which the level of digital health solutions usage varies. Most of the studies were conducted in the United States. Thus, the scope of the studies might not have provided generalized results about web-based patient-professional communication because health care systems and digital solutions vary between different countries.

### Conclusions

Health care professionals had both positive and negative experiences related to web-based patient-professional communication. The positive experiences were most commonly related to the patients and patient outcomes, such as learning more about patients’ situations and having better patient engagement. The negative experiences were related to aspects such as the additional workload on health care professionals, deficiencies in communication, patients’ false interpretations, and the suitability of patient portals for communication. Negative experiences of health care professionals related to the use of patient portals seemed to be associated to the poor functionality of the portals and insufficient training and resourcing.

## Abbreviations

EHRs: electronic health records
JBI: Joanna Briggs Institute
PRISMA: Preferred Reporting Items for Systematic Reviews and Meta-Analyses
